# Supplementary material for: Current state and open problems in universal differential equations for systems biology
Source: NPJ Syst Biol Appl. 2025 Aug 30;11:101. doi: 10.1038/s41540-025-00550-w (PMC12398592; doi:10.1038/s41540-025-00550-w)
Supplement: Supplementary file 1 — Supplementary Information [file 41540_2025_550_MOESM1_ESM.pdf]

Supplemental Material: Current state and open problems in universal differential equations for systems biology

1 Glycolysis

The estimated mechanistic parameters, their bounds and the scale used for optimisation is listed in Table 1.

**Parameter name**	**Scale**	**Bounds**	**True value**	**UDE**
J_0	log10	1e-1, 1e2	2.5	Yes
k_1	log10	1, 1e3	100	Yes
k_2	log10	1e-1, 1e2	6	Yes
k_3	log10	1e-1, 1e2	16	Yes
k_4	log10	1, 1e3	100	Yes
k_5	log10	1e-1, 1e2	1.28	No
k_6	log10	1e-1, 1e2	12	Yes
k_{ex}	log10	1e-1, 1e2	1.8	Yes
kappa	log10	1e-1, 1e2	13	Yes
K_1	log10	1e-2, 1e1	0.52	Yes
N	log10	1e-1, 1e2	1	Yes
A	log10	1e-1, 1e2	4	Yes
phi	log10	1e-2, 1e1	0.1	Yes
sd N_2 (5% noise)	linear	0, 0.025	0.00606935	Yes
sd N_2 (10% noise)	linear	0, 0.05	0.0121387	Yes
sd N_2 (20% noise)	linear	0, 0.1	0.0242774	Yes
sd N_2 (35% noise)	linear	0, 0.2	0.04248545	Yes
sd A_3 (5% noise)	linear	0, 0.4	0.0929227	Yes
sd A_3 (10% noise)	linear	0, 0.8	0.1858454	Yes
sd A_3 (20% noise)	linear	0, 1.6	0.3716908	Yes
sd A_3 (35% noise)	linear	0, 3.2	0.6504589	Yes

Table 1: Mechanistic parameters with their optimisation scale, bounds, true value, and whether it was optimised in the UDE as well.

A dense data set with altogether 61 data points was generated and subsampled to create sparse data sets with 46, 31, 16 and 8 data points in total. All data sets were noise-corrupted with additive normally-distributed noise and standard deviation of 5%, 10%, 20% or 35% of the nominal values. A dense ($\Delta t = 0.01$) and noise-free simulation in $t \in [1.5, 5]$ serves as the test set for all training data sets.

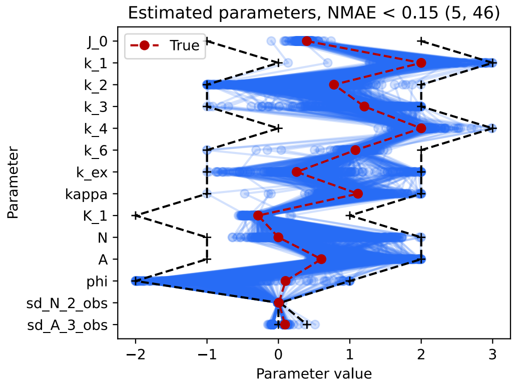

Fig. 1: Parameter vectors of the successful fits for the dataset with 5% noise and 46 data points.

Reg. λ	UDE ID	Symbolic regression	Complexity
0	129253	$0.03054 \cdot S_3 - 3.229$	5
1e-3 – 1e-2	129393	$-107.3 \cdot (S_3 \cdot S_2) - 3.252$	7
1e-2 – 1e-1	126616	$-8.778 \cdot {S_3}^2 - 3.054$	7
0.1 – 1	128044	$-1.618 \cdot S_4 - 3.067$	7
1 – 10	124014	$-8.057 \cdot S_3 - 2.527$	7

Table 2: Symbolic regression results for the glycolysis model, by regularisation strength λ. True solution: $-1.28A_3$

.

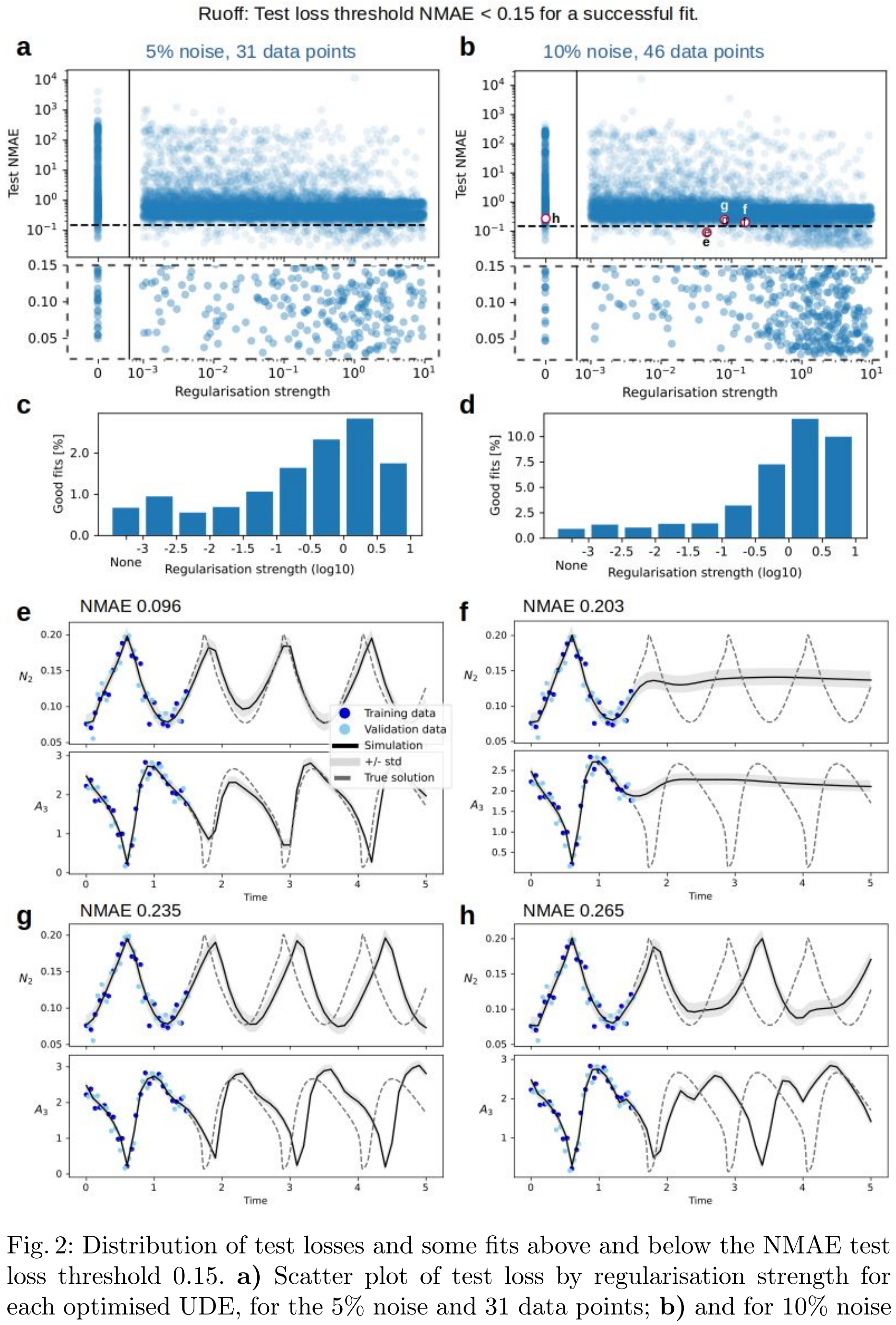

Fig. 2: Distribution of test losses and some fits above and below the NMAE test loss threshold 0.15. **a)** Scatter plot of test loss by regularisation strength for each optimised UDE, for the 5% noise and 31 data points; **b)** and for 10% noise and 46 data points. Additionally, the location of the UDEs from panels e-h are labeled. **e)** Predictions for a successful fit because the test loss fall below the threshold. **f-g)** predictions from failed models.

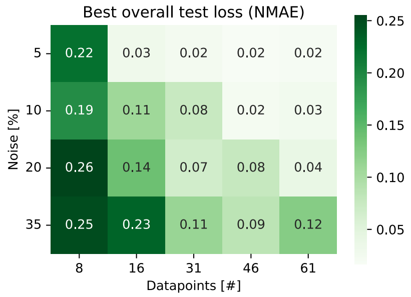

Fig. 3: The best test loss (NMAE) by data setting.

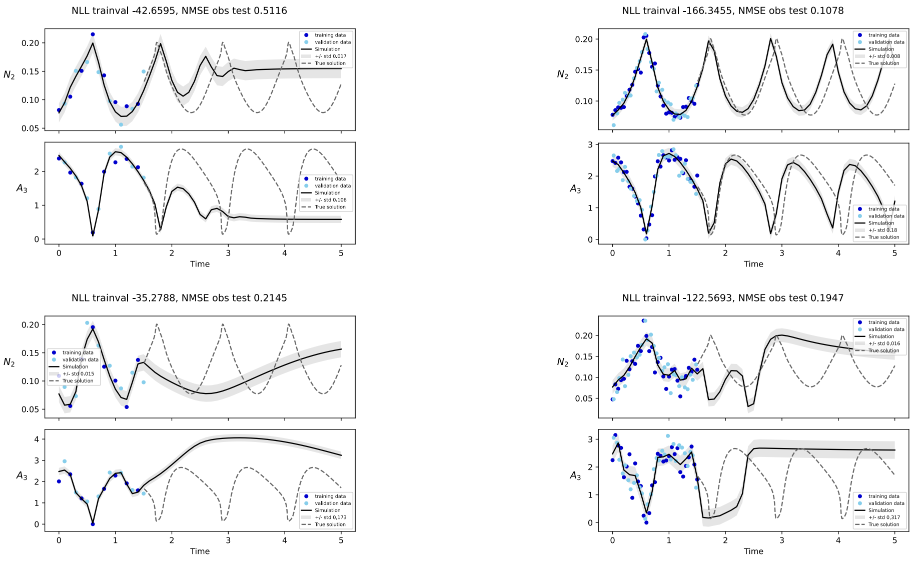

Fig. 4: Best fits from glycolysis model on datasets with 16 (left column) and 61 (right column) data points per observable; and lower noise (upper row, 10%) or higher noise (lower row, 20%).

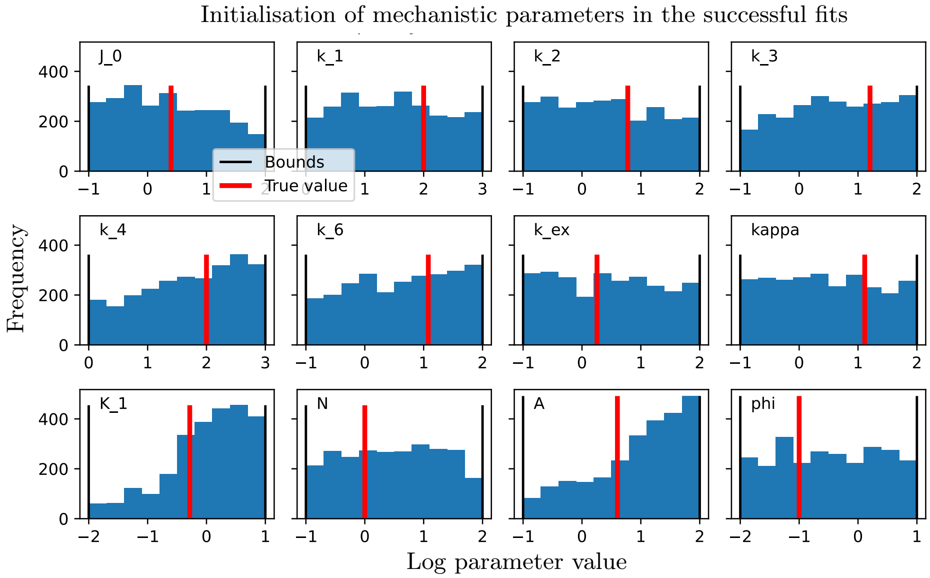

Fig. 5: Initial values for the mechanistic parameters, for all successfully fitted models (test loss < 0.15).

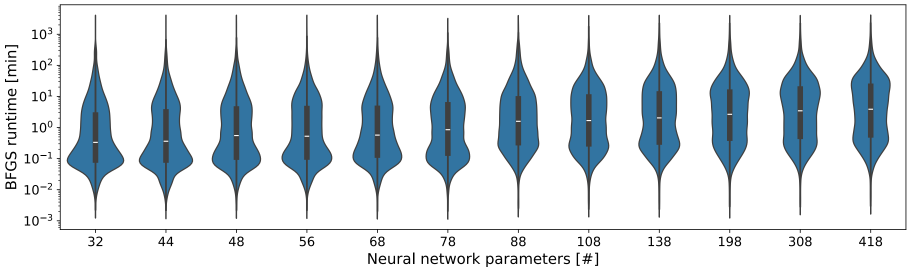

Fig. 6: Distribution of BFGS runtime by number of ANN parameters (weights + biases).

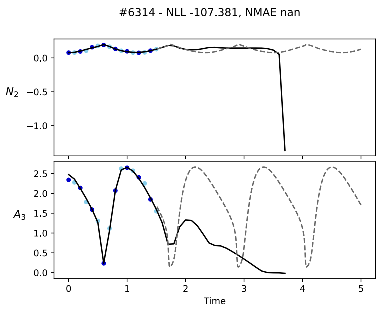

Fig. 7: UDE with best training loss that was trained on 16 data points with 5% noise without regularisation.

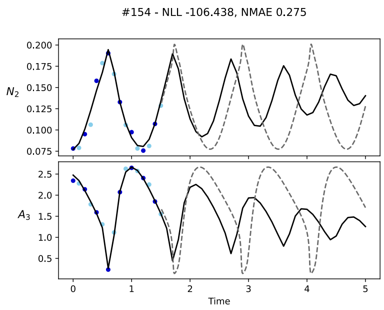

Fig. 8: UDE with best training loss that was trained on 16 data points with 5% noise with regularisation strength $1 < \lambda < 10$.

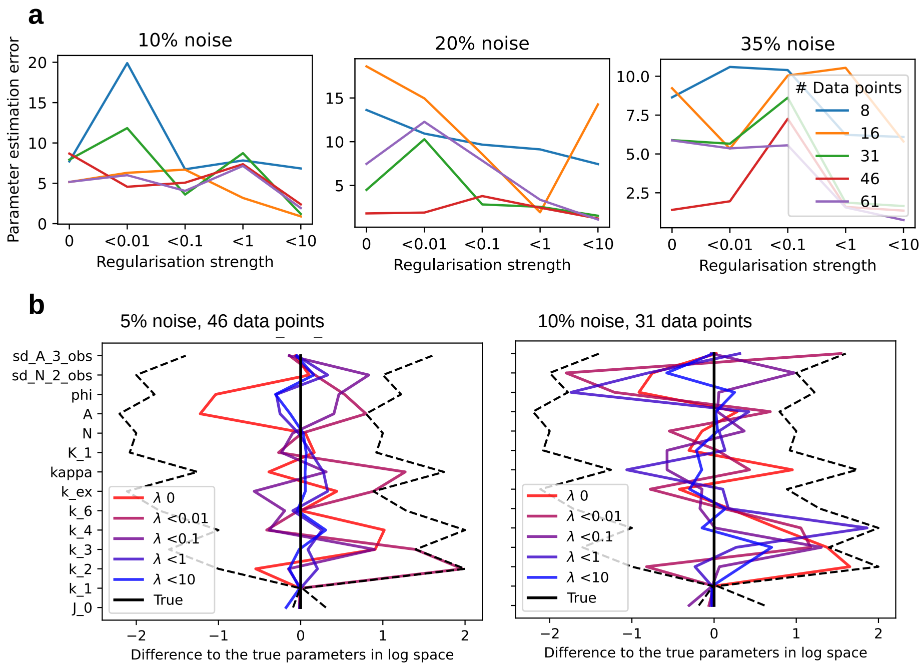

Fig. 9: **(a)** Parameter estimation error for the best UDEs by regularisation strength, shown for different data sets. **(b)** Parameter error per mechanistic parameter, i.e. difference to the true parameter, depending on the regularisation strength, show for two data sets.

2 STAT5 dimerisation

The estimated mechanistic parameters, their bounds and the scale used for optimisation is listed in Table 3. Unless indicated otherwise, all mechanistic parameters were used in all UDE *Scenarios*.

Parameter name	Scale	Bounds	Reference value	Comment
Epo_degradation_BaF3	log10	1e-5, 1e5	0.026982514033029	
k_exp_hetero	log10	1e-5, 1e5	1.00067973851508e-5	
k_exp_homo	log10	1e-5, 1e5	0.006170228086381	
k_imp_hetero	log10	1e-5, 1e5	0.0163679184468	
k_imp_homo	log10	1e-5, 1e5	97749.3794024716	
k_phos	log10	1e-5, 1e5	15766.5070195731	
sd pSTAT5A_rel	log10	1e-5, 1e5	3.85261197844677	
sd pSTAT5B_rel	log10	1e-5, 1e5	6.59147818673419	
sd rSTAT5A_rel	log10	1e-5, 1e5	3.15271275648527	
k_exp_aug_A	log10	1e-5, 1e5	0	Only used in UDE *Scenario 4*
k_exp_aug_AB	log10	1e-5, 1e5	0	
k_exp_aug_B	log10	1e-5, 1e5	0	

Table 3: STAT5 model mechanistic parameters with their optimisation scale, bounds, and reference value from the original publication [1].

Framework	Solver	Adaptive	Simulation Time	Simulation Success
torchdiffeq	8th-order Runge-Kutta of Dormand-Prince-Shampine	Yes	$>$ 12h	No (timeout)
torchdiffeq	5th-order Runge-Kutta of Dormand-Prince-Shampine	Yes	$>$ 12h	No (timeout)
torchdiffeq	3rd-order Runge-Kutta of Bogacki-Shampine	Yes	$>$ 12h	No (timeout)
torchdiffeq	2nd-order Runge-Kutta-Fehlberg	Yes	$>$ 12h	No (timeout)
torchdiffeq	2nd-order Runge-Kutta	Yes	$>$ 12h	No (timeout)
SciML	KenCarp4	Yes	0.0003 s	Yes
SciML	Tsit5	Yes	0.303 s	Yes

Table 4: Simulation results for all available torchdiffeq solvers and selected SciML solvers when simulating the original STAT5 dimerisation problem with true parameter values.

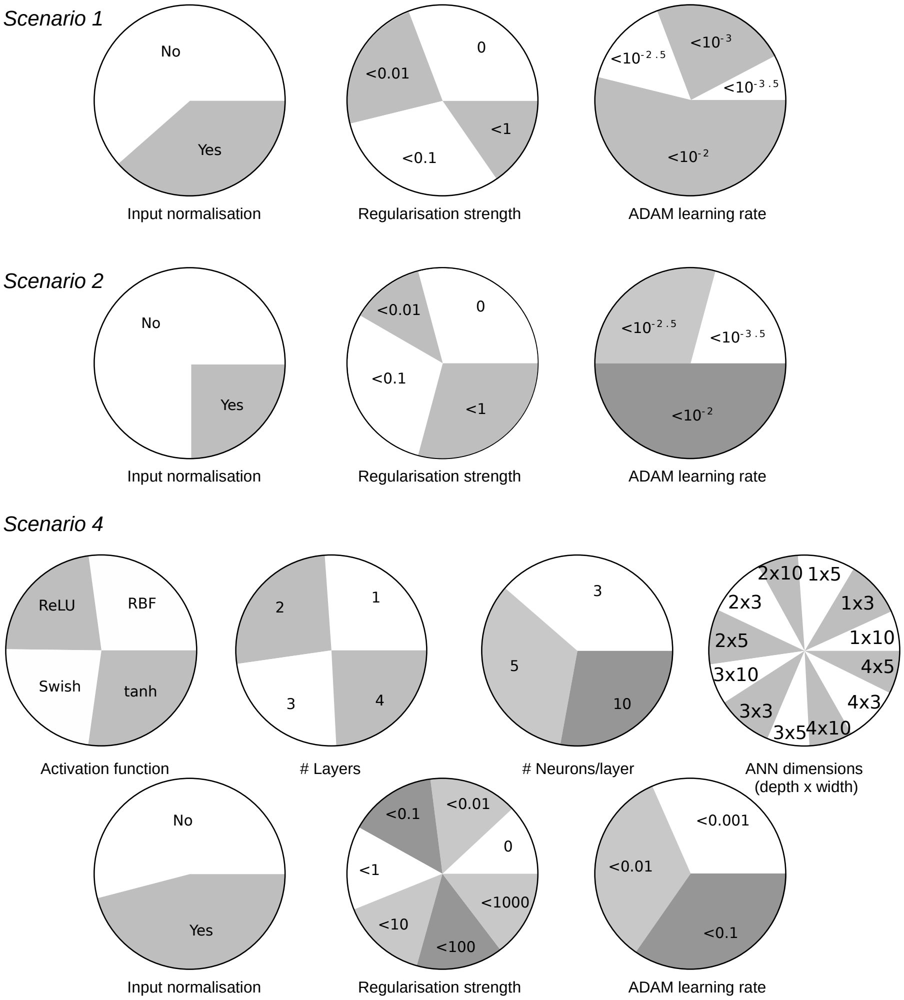

Fig. 10: For all successful fitted models of *Scenario 1, 2, 4*: Each pie represents one hyperparameter and the pie's fractions correspond to the number of successful fits with a specific setting. The continuously sampled hyperparameters (learning rate, regularisation strength) were binned, and the labels show the upper bound. Number of successful fitted models: 13 (*Scenario 2*), 24 (*Scenario 2*), 22719 (*Scenario 4*).

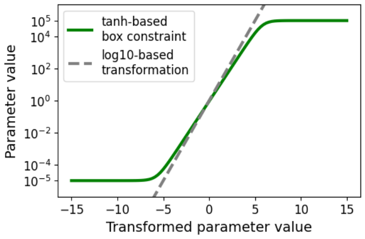

Fig. 11: Tanh-based parameter transformation for $b_l = 10^{-5}$ and $b_u = 10^5$ in comparison to a log10 transformation.
